# A Comparation Between Frame-Based and Robot-Assisted in Stereotactic Biopsy

**DOI:** 10.3389/fneur.2022.928070

**Published:** 2022-07-18

**Authors:** Yue Hu, Pu Cai, Huawei Zhang, Aihemaitiniyazi Adilijiang, Jun Peng, Yun Li, Shanli Che, Fei Lan, Changqing Liu

**Affiliations:** ^1^Department of Neurosurgery, Sanbo Brain Hospital, Capital Medical University, Beijing, China; ^2^Department of Neurosurgery, Chongqing Sanbo Jiangling Hospital, Chongqing, China; ^3^Center of Epilepsy, Beijing Institute for Brain Disorders, Beijing, China

**Keywords:** intracranial lesions, robot-assisted surgery, brain biopsy, frameless stereotactic biopsy, SINO robot

## Abstract

**Introduction:**

Frame-based stereotactic biopsy is well-established to play an essential role in neurosurgery. In recent years, different robotic devices have been introduced in neurosurgery centers. This study aimed to compare the SINO surgical robot-assisted frameless brain biopsy with standard frame-based stereotactic biopsy in terms of efficacy, accuracy and complications.

**Methods:**

A retrospective analysis was performed on 151 consecutive patients who underwent stereotactic biopsy at Chongqing Sanbo Jiangling Hospital between August 2017 and December 2021. All patients were divided into the frame-based group (*n* = 47) and the SINO surgical robot-assisted group (*n* = 104). The data collected included clinical characteristics, diagnostic yield, operation times, accuracy, and postoperative complications.

**Results:**

There was no significant difference in diagnostic yield between the frame-based group and the SINO surgical robot-assisted group (95.74 vs. 98.08%, *p* > 0.05). The mean operation time in the SINO surgical robot-assisted group was significantly shorter than in the frame-based group (29.36 ± 13.64 vs. 50.57 ± 41.08 min). The entry point error in the frame-based group was significantly higher than in the robot-assisted group [1.33 ± 0.40 mm (0.47–2.30) vs. 0.92 ± 0.27 mm (0.35–1.65), *P* < 0.001]. The target point error in the frame-based group was also significantly higher than in the robot-assisted group [1.63 ± 0.41 mm (0.74–2.65) vs. 1.10 ± 0.30 mm (0.69–2.03), *P* < 0.001]. Finally, there was no significant difference in postoperative complications between the two groups.

**Conclusion:**

Robot-assisted brain biopsy becomes an increasingly mainstream tool in the neurosurgical procedure. The SINO surgical robot-assisted platform is as efficient, accurate and safe as standard frame-based stereotactic biopsy and provides a reasonable alternative to stereotactic biopsy in neurosurgery.

## Introduction

In recent years, the diagnostic yield of intracranial lesions has substantially improved due to the development of neuroimaging (1). In clinical practice, tissue biopsy is often required to confirm the diagnosis and determine the treatment regimen given the heterogeneity surrounding clinical characteristics and imaging features (2–4). Frame-based stereotactic biopsy has long been the gold standard for diagnosing intracranial lesions due to its efficacy and safety (1, 5, 6). However, traditional stereotaxy has disadvantages, such as cumbersome stereotactic frames, space requirements, and limited scope of application in children (7, 8).

Over the past 4 decades, the implementation of robotic technology has transitioned from the industrial sector to become an essential tool in surgical practice (9, 10). The first robot-assisted brain biopsy was conducted on a 52-year-old man at the Memorial Medical Center (11). Subsequently, robot-assisted stereotactic brain biopsy has become a mainstream tool in the neurosurgical armamentarium (12, 13) since it meets safety, flexibility, versatility, accuracy, and stability requirements. The Chongqing Sanbo Jiangling Hospital has applied the Chinese SINO surgical robot for stereotactic biopsy since October 2019. This study compared the surgical robot-assisted frameless brain biopsy with standard frame-based stereotactic biopsy in terms of efficacy, accuracy, and safety.

## Materials and Methods

### Patient Population

This study was approved by the ethics committee of Chongqing Sanbo Jiangling Hospital. The inclusion criteria were as follows: (1) Patients with imaging evidence of intracranial lesions, for which no definite diagnosis could be established. (2) Lesions in the deep brain or eloquent areas. (3) Imaging evidence of multifocal or diffuse lesions not suitable for resection. (4) Patients unfit for surgery requiring pathological evidence to guide radiotherapy or chemotherapy. The exclusion criteria consisted of (1) No definite lesions on imaging. (2) Patients with severe coagulopathy or unstable vital signs. (3) The patient or his family members refused the biopsy. The surgical approach was decided by the neurosurgical team and the patient or the patient's guardian. Finally, a total of 151 patients were enrolled in this study between August, 2017 and December, 2021. Forty-seven patients underwent Leksell (Elekta Ltd., Stockholm, Sweden) frame-based brain biopsy, and 104 patients underwent SINO (Sinovation Medical Technology Co., Ltd, Beijing, China) robot-assisted brain biopsy. All surgeries were performed by the same neurosurgeon, Professor Changqing Liu, Department of Neurosurgery, Chongqing Sanbo Jiangling Hospital.

### Data Collection

The demographic and clinical characteristics were retrospectively analyzed, including patient age, gender, locations of lesions, etc. For the histological diagnosis, the World Health Organization classification amended in 2016 was used (2). The operation time was used to assess the efficiency of the surgery, but the preoperative CT scan and registration time were not included. The entry point error (EPE) and target point error (TPE) represented the accuracy of the surgical procedure. The EPE was defined as the distance between the planned and actual entry points. We measured the EPEs as previously described by Dlaka et al. (14). Besides, the EPE was measured on the cranial bone based on postoperative CT. The TPE was defined as the distance between the position of the actual operation that was estimated by the coordinates of the center of the biopsy site and the corresponding position of the planned surgical target. Assessment of TPE was conducted by merging the postoperative CT data with the preoperative dataset. Postoperative complications were used to reflect the safety of the procedure. The main complication was bleeding at the biopsy sites and/or along the stereotactic trajectory.

### Presurgical Planning and Surgical Device

**Figure 1:**
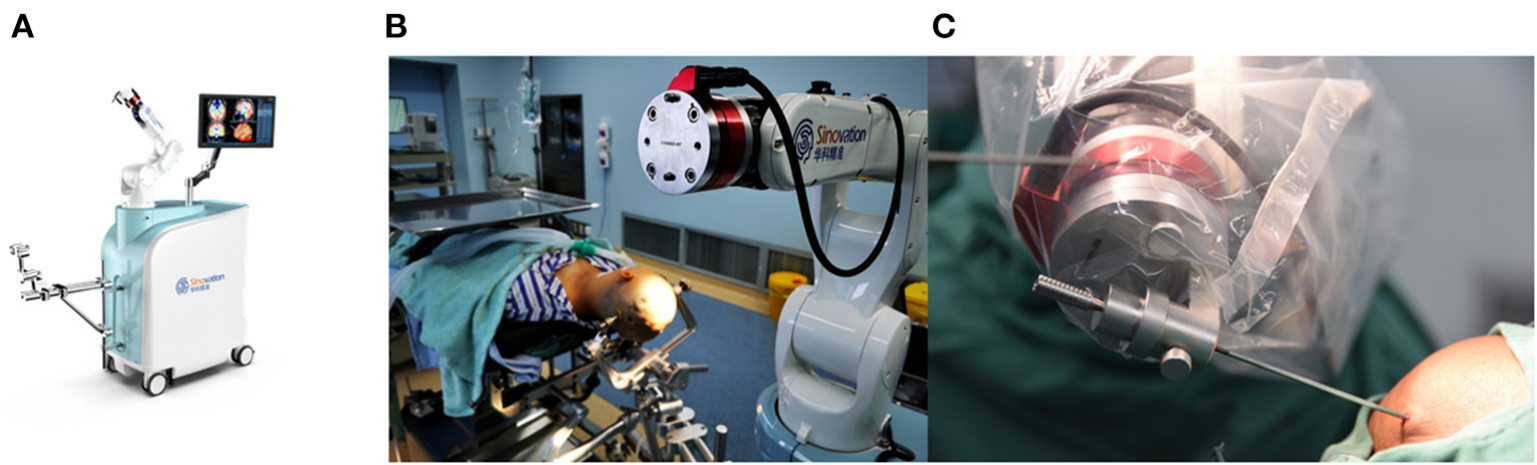
**(A)** The SINO surgical robot. **(B,C)** The robot for brain biopsy.

**Figure 2:**
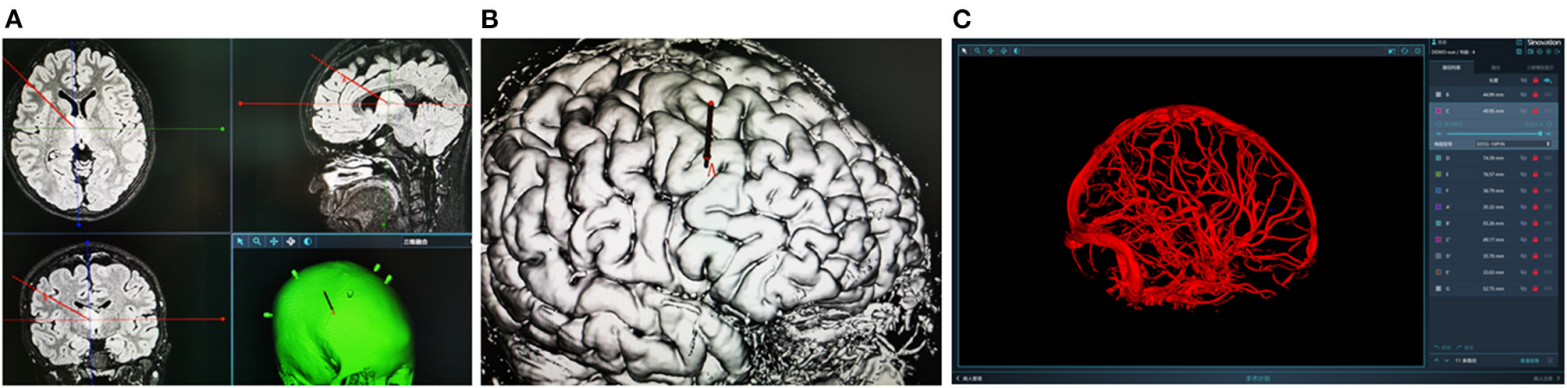
**(A,B)** Design the stereotactic trajectory on the Sinoplan software. **(C)** Three-dimensional (3D) visualization technology of craniocerebral vascular.

Presurgical magnetic resonance imaging (Siemens 1.5T, sequence 3D dimension (3D)-T1 with gadolinium, 1.5 mm thick, and T2 flair sequence) was performed the day before the procedure. Other MRI sequences were used depending on the lesions. The images were imported into the surgical planning system. Then the stereotactic trajectory was designed to avoid important tissue structures such as blood vessels, sulci, and eloquent areas as much as possible. The merged image was checked and corrected manually by an expert neurosurgeon before the surgery. SR1 is a robotic arm system equipped with six degrees of freedom to ensure the robot's flexibility. The robot device and working platform are shown in Figures 1, 2, respectively. In addition, to ensure operation safety, the system is also equipped with an automatic touch avoidance function (15).

### Surgical Procedure

For the frame-based biopsy, we fixed the Leksell stereotactic frame on the patient's head under local anesthesia before the operation. Then, a CT scan (Simens CT, thickness of cutting 1 mm) was performed. The reference CT scan was uploaded to the workstation and merged with the preoperative MR images. Reconstructed images along the planned trajectory were used to verify that the stereotactic trajectory was satisfactory and reduce the risk of hemorrhage during the surgical procedure. The surgical procedure was performed under general anesthesia. A burr hole was made with a 3.0 mm diameter drill. Then, the surgeon cauterized and perforated the dura using a monopolar coagulation needle. After ensuring no active bleeding occurred, a biopsy needle was inserted into the target lesion. At least four specimens were collected at the target site.

For the robot-assisted biopsy, we placed at least five bone fiducials on the patient's head in the neurosurgical ward on the day of surgery. The skull positioning nails (4 mm in diameter and 5 mm in length) can penetrate through 2–3 mm of the skull. Then the patients went through the same processes, such as CT scan, images reconstructed, etc. In the operation room, the head of the patient was fixed in a Mayfield head holder connected to the robot. Patients were placed in a supine or lateral prone position according to the lesion's location. During the operation, the puncture point and puncture depth were conducted by the Sinovation software system and a mechanical arm with an error margin <0.35 mm. After skin asepsis and placement of surgical drapes, the robotic arm was positioned in line with the trajectory. A 3.0 mm drill and a coagulation probe (Sutter, 1.5 mm) (to coagulate the dura) were installed on the instrument holder. The biopsy needle (190 mm, sample pane of 10 mm, lateral opening) was passed from the robot arm to the desired target. Core biopsies of lesion tissue were acquired using a negative pressure suction technique. The needle was sequentially rotated to obtain 4 separate specimens at each of the desired sites.

**Figure 3:**
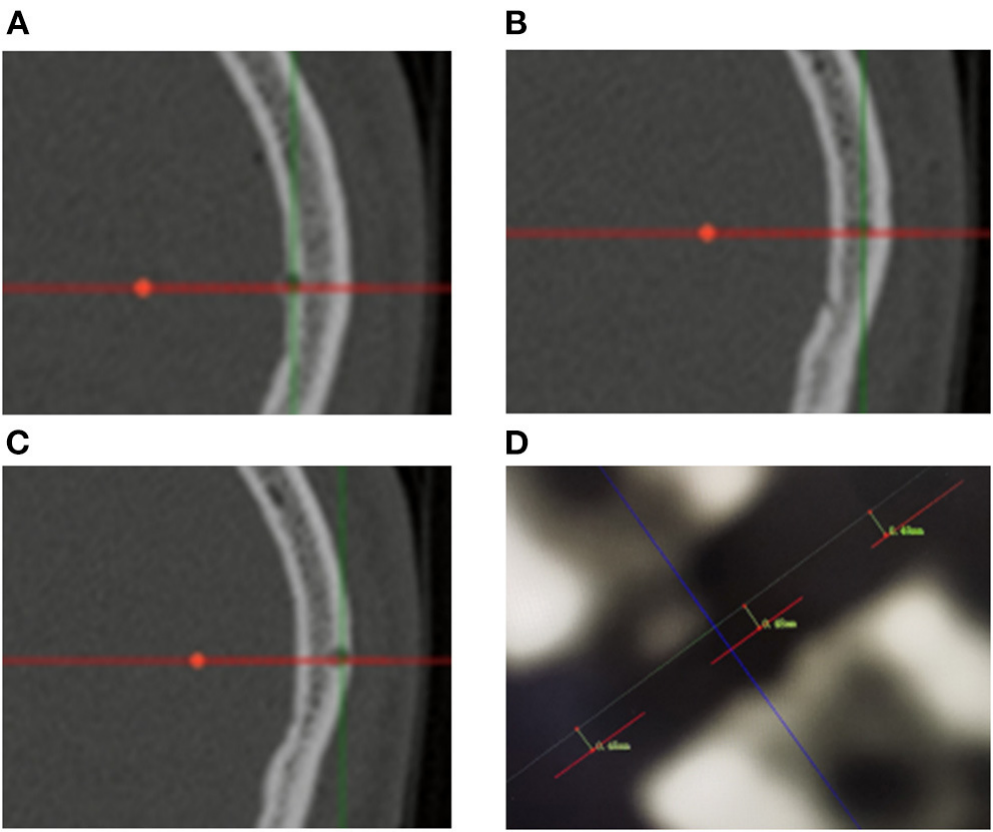
**(A–C)** Measurement of entry point error based on postoperative CT scanning. The red lines represent the biopsy trajectory planned preoperatively. Bone defects represent the actual biopsy trajectory of the operation. The EPEs are computed as the average of the measurement of the inner part, midpoint and outer part of the cranial bone. **(D)** The picture shows the measurement results of one of the patients. The EPEs were 0.48, 0.48, and 0.49 mm, respectively.

**Figure 4:**
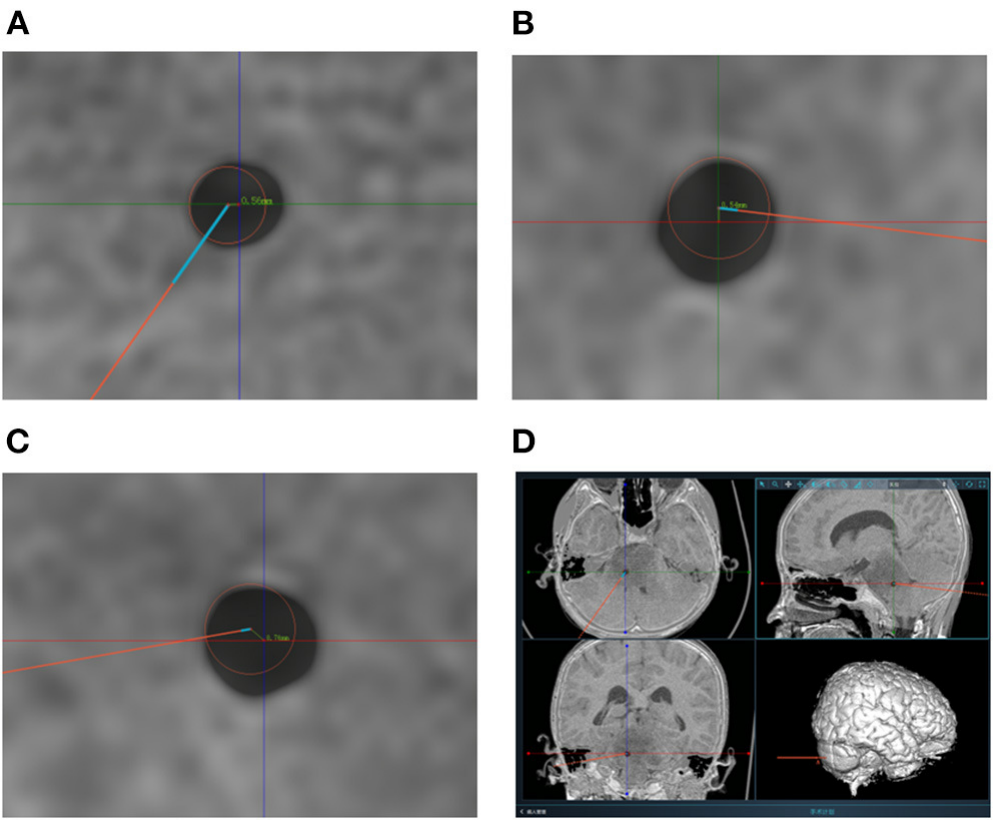
**(A–C)** Measurement of target point error based on postoperative CT scanning. The centers of the red circles represent the target points planned preoperatively. The TPEs are computed based on the errors from axial position, coronal position and sagittal position respectively. **(D)** The picture shows the measurement of one of the patients. And the TPEs were 0.52, 0.54, and 0.76 mm, respectively. The actual TPE was 1.07 mm.

The lesion biopsies were performed at different sites to minimize the sampling error. Biopsy specimens were analyzed in the pathology laboratory of Sabo Brain Hospital, Capital Medical University. A control CT scan was systematically performed on day 0 after surgery to ensure no bleeding along the route or at biopsy sites and postsurgical swelling. The measurements of EPE and TPE based on the fusion of postoperative CT to the preoperative dataset are shown in Figures 3, 4.

### Statistical Analysis

All statistical analyses were performed using SPSS (IBM SPSS Statistics, version 26.0.; IBM Corporation, USA). The categorical data were described as frequencies and percentages. The continuous variables were presented as mean ± standard deviation and ranges. The intergroup comparisons were assessed using the c^2^ test or the non-parametric Mann-Whitney *U*-test. A *P*-value <0.05 was statistically significant.

## Results

### Demographic Characteristics

In the frame-based group, 47 patients (28 men) with a mean age of 49.19 ± 15.76 years (18.0–82.0) underwent stereotactic biopsy; the lesions were solitary and supratentorial in 32 and 38 patients, respectively. In the robot-assisted group, 104 patients (62 men) with a mean age of 45.30±19.15 years (1.2–82.0) underwent a stereotactic frameless biopsy; the lesions were solitary and supratentorial in 67 and 78 patients (Table 1). Most lesions were located in the frontal lobe (27.8%). Extralobar lesions were primarily located in the brain stem (17.9%) (Table 2). There was no significant difference in demographic characteristics between the two groups. Detailed information is provided in Tables 1, 2.

**Table 1:** Summary of the demographic and surgical characteristics in patients.

**Characteristics**	**Frame-based group (*n* = 47)**	**Robot-assisted group (*n* = 104)**	***P***
Sex			0.996
Male	28	62	
Female	19	42	
Age at surgery	49.19 ± 15.76 (18.0–82.0)	45.30 ± 19.15 (1.2–82.0)	0.239
Distribution			0.661
Solitary	32	67	
Multiple	15	37	
Side			0.297
Left	20	32	
Right	16	34	
Midline	8	22	
Bilateral	3	16	
Location			0.430
Supratentorial	38	78	
Infratentorial	9	26	
Entry point error (mm)	1.33 ± 0.40 (0.47–2.30)	0.92 ± 0.27 (0.35–1.65)	<0.001^a^
Target point error (mm)	1.63 ± 0.41 (0.74–2.65)	1.10 ± 0.30 (0.69–2.03)	<0.001^a^
Operation time^b^ (min)	50.57 ± 41.08 (16.00–210.00)	29.36 ± 13.64 (10.00–75.00)	<0.001^a^
Stereotactic trajectory^c^	1.36 ± 0.57 (1–3)	1.30 ± 0.48 (1–3)	0.478
Biopsy site	2.21 ± 1.44 (1–6)	1.91 ± 0.93 (1–6)	0.128
Complications	5/47 (9.76%)	9/104 (8.65%)	0.697

*^a^ P <0.05. ^b^ Time of preoperative preparation was not included. ^c^ In order to reduce the sampling error, we may biopsy the lesion with different stereotactic trajectories*.

**Table 2:** Localization of target lesion in 151 stereotactic biopsies.

**Location**	**Value**	**Percentage (%)**
Frontal lobe	42	27.8
Temporal lobe	15	9.9
Parietal lobe	20	13.2
Occipital lobe	4	2.6
Insula	3	2.0
Basal ganglia	14	9.3
Thalamus	10	6.6
Brain stem	27	17.9
Corpus callosum	7	4.6
Cerebellum	7	4.6
Ventricle	2	1.3

### Histological Diagnosis

Histological findings in the frame-based group included glial or glioneuronal tumors (*n* = 28), lymphoma (*n* = 7), cerebral infarction (*n* = 2), metastasis (*n* = 4), inflammatory lesion (*n* = 2) and Syphilis (*n* = 1). In the robot-assisted group, histological findings included glioma (*n* = 59), lymphoma (*n* = 15), cerebral infarction (*n* = 6), inflammatory lesions (*n* = 6) and others (*n* = 11). In addition, non-specific findings were found in 2 case in the frame-based group and 2 cases in the robot-assisted group (Table 3). There was no significant difference in diagnostic yield between the frame-based and robot-assisted groups (95.74 vs. 98.08%, *P* = 0.409).

**Table 3:** Histologic diagnosis in 151 stereotactic biopsies.

**Histology**	**Frame-based group (*n* = 47)**	**Robot-assisted group (*n* = 104)**	***P***
Diagnostic yield	45/47 (95.74%)	102/104 (98.08%)	0.409
Glioma	28	59	
Lymphoma	7	15	
Cerebral infarction	2	6	
Metastases	4	5	
Inflammatory lesion	2	6	
Granuloma	-	2	
Hemorrhage necrosis	1	2	
Gliosarcoma	-	1	
Histiocytosis	-	1	
Syphilis	1	1	
Cavernous hemangioma	-	1	
Multifocal leukoencephalopathy	-	1	
Gliosis	-	1	
Mitochondrial encephalopathy	-	1	
Unspecific findings	2	2	

### Operation Time

The mean operation time in the SINO surgical robot-assisted group was significantly shorter than in the frame-based group (29.36 ± 13.64 vs. 50.57 ± 41.08 min), while the number of biopsy sites was comparable between both groups (1.91 ± 0.93 vs. 2.21 ± 1.44, *P* = 0.128).

### Surgical Accuracy

**Figure 5:**
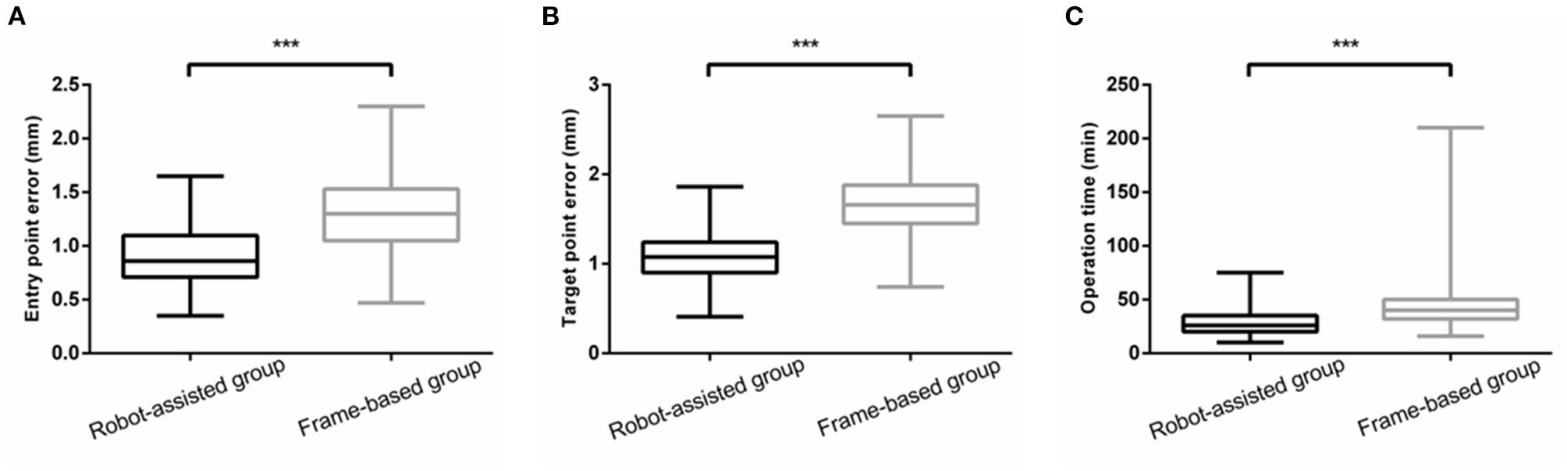
**(A,B)** The EPE and TPE of robot-assisted group were significantly less than that of frame-based group. **(C)** There was a significant reduction in operation time ***means *P* < 0.001.

To compare the accuracy of the two kinds of stereotactic biopsies, we measured the target point error of the two groups. The entry point error in the frame-based group was significantly higher than in the robot-assisted group [1.33 ± 0.40 mm (0.47–2.30) vs. 0.92 ± 0.27 mm (0.35–1.65), *P* < 0.001]. The target point error was significantly greater in the frame-based group than in robot-assisted group [1.63 ± 0.41 mm (0.74–2.65) vs.1.10 ± 0.30 mm (0.69–2.03), *P* < 0.001]. Based on our experience and findings of previous studies, robot-assisted stereotaxy is relatively more accurate. Further details are provided in Figure 5.

### Complications

The main complications were hemorrhage and clinical impairment. Hemorrhage was observed on CT scan in the frame-based group (*n* = 4) and robot-assisted group (*n* = 6). In these 10 cases, bleeding was restricted within the lesion and not observed along the biopsy trajectory. No patient exhibited clinical deterioration related to bleeding complications. For patients with hemorrhage within the lesion and brain stem edema, mannitol and steroids were used depending on the patient's specific needs to decrease the mass effect of the hematoma and edema. One case in the frame-based group exhibited suspicious focal seizures characterized by involuntary shaking of one hand that resolved spontaneously within seconds. Three cases of brainstem lesions in the robot-assisted group exhibited clinical impairment, including muscle weakness and dysphagia following surgery for brain glioma. No cases of infection and mortality were observed. Moreover, there was no significant difference in the incidence of complications between both groups (*P* = 0.697).

## Discussion

Stereotactic brain biopsy is well-established as a safe and effective way to diagnose brain occupying lesions that can assist in the diagnostic workup to ensure that patients receive optimal therapy (3, 8, 16). Frame-based stereotactic biopsy has long been the gold standard for diagnosing intracranial lesions (17, 18). In recent years, robot-assisted frameless stereotactic biopsy has gradually replaced frame-based stereotactic biopsy in large neurosurgical centers, given their refined safety, flexibility, versatility, and accuracy. This study showed no significant differences in either complications or diagnostic yield between robotic and stereotactic biopsies. However, a significant reduction in operation time was associated with robot-assisted biopsy compared to frame-based biopsy. Besides, robotic biopsy exhibited higher accuracy rates.

### Efficacy

It is widely acknowledged that the efficiency of biopsy of brain lesions is mainly reflected by higher diagnosis yield and shorter operation time. In this regard, asystematic review by Dlaka et al. (14) reported a diagnostic yield ranging from 75 to 100% for robot-assisted stereotactic biopsy (14). An increasing body of evidence suggests that the diagnostic yield of classic frame-based stereotactic biopsies ranges from 81.3 to 99.2% (1, 13, 19–26). Yi et al. reported no significant difference in diagnostic yield between frame-based biopsies and frameless biopsies (96.9 vs. 91.8%). In a retrospective analysis (19) by Wu et al. (1) no significant difference in diagnostic yield was found (91.4 vs. 93.5%). Consistently, in the present study, no significant difference in diagnostic yield was observed between the frame-based group and the robot-assisted group (95.74 vs. 98.08%, *P* = 0.328).

The operation time for robot-assisted biopsies reported in our study is consistent with the literature (3, 27). Compared with traditional stereotactic biopsy, the operation time was shorter with robot-assisted biopsy attributed to the fact that robot-assisted stereotactic biopsy only required importing data of the designed trajectory into the operating platform. In contrast, the frame-based biopsy required checking the parameters of each target which can be time-consuming especially for multifocal intracranial lesions since the Leksell frame must be loaded and unloaded. Moreover, a frame-based biopsy requires cooperation among surgeons. Indeed, the frame-based biopsy is more time-consuming if the preoperative preparation is considered. The frame must be fixed to the patient's skull for a CT scan on the day of operation and then returned to the operating room to wait for the parameters of the biopsy site. In contrast, the surgeon only needs to design the stereotactic trajectory in the supporting software for robot-assisted biopsy according to the preoperative MRI image. Before surgery, the results are imported into the robot system and processed which only takes a few minutes. Naros et al. (28) reported 42 biopsy surgeries using ROSA non-frame robot with an operation time of 25 ± 15 min. In study by Dlaka et al. (14) where brain biopsies with RONNA G4 were conducted (*n* = 32), the average operation duration was 64.62 ± 19.05 min substantiating the SINO robot-assisted biopsy procedure is comparable to other existing robots in terms of efficiency with an operation time of 29.36 ± 13.64 min. It should be pointed out that no learning curve was associated with using the SINO surgical robot in this study. Although the SINO surgical robot was introduced to hospitals in October 2019, neurosurgeons were already trained to operate it.

### Accuracy

Ample evidence suggests that the accuracy of the stereotactic device is essential during biopsy surgery (27, 29). However, few studies compared frame-based biopsy with robot-assisted biopsy for accuracy of entry point error or target point error. According to the literature, the Leksell frame-based stereotaxy is accurate, with a reported target point error ranging from 1.7 to 2.5 mm (26, 30). In the present study, the entry point error in the frame-based group was significant higher than in thr robot-assisted group [1.33 ± 0.40 mm (0.47–2.30) vs. 0.92 ± 0.27 mm (0.35–1.65), *P* < 0.001]. Moreover, the target point error is another indicator of the accuracy of stereotactic neurosurgery. In the present study, the target point error in the frame-based group was significantly higher than in the robot-assisted group [1.63 ± 0.41 mm (0.74–2.65) vs. 1.10 ± 0.30 mm (0.69–2.03), *P* < 0.001].

Overwhelmng evidence substantiates that robotic technology has very high accuracy. Liu et al. (3) reported an entry point error of 0.99 ± 0.24 mm (ranging from 0.56 to 1.73 mm) and target point error of 1.13 ± 0.30 mm (ranging from 0.57 to 1.78 mm) after conducting 700 biopsy surgeries using the Remebot device. Dlaka et al. (14) reported a target point error of 1.95 ± 1.11 mm and an entry point error of 1.42 ± 0.74 mm for 32 brain biopsies with RONNA G4 system. Besides, Alessandro De Benedictis et al. (10) reported an entry point error of 1.59 ± 1.1 mm and a target point error of 2.22 ± 1.71 mm for 36 patients that underwent electrode implantation performed by ROSA. Minchev et al. (31) conducted a median real target error of 1.3 mm at entry and 0.9 mm at the target point for 25 patients with iSYS1 robot. In addition, the median target point localization error with the Neuromate root (Renishaw, Gloucestershire, UK) has been reported to be 2.7 mm (range from 0.5 to 4.2 mm) (32). Other kinds of robots with high accuracy include the Surgiscope (Elekta AB, Stockholm, Sweden) (21), MKM (Carl Zeiss Co., Oberkochen, Germany) (33), and Neuromate (Renishaw Mayfield SA, Nyon, Switzerland) (18). The SINO surgical robot is as accurate as other robots reported in the literature, although randomized controlled trials have not been conducted. Moreover, bone-implanted fiducial markers were adopted for registration during the robot-assisted surgical procedure. Consistently, Machetanz et al. (29) reported that bone-implanted fiducials for landmark registration were accurate. Last but not least, frame-based biopsy requires meticulous manual adjustment for each biopsy site from the planning software to the frame, which may increase the risk of errors.

### Safety

The main complication of stereotactic biopsy is hemorrhage, with a reported incidence ranging from 0 to 14.6% (3, 10, 18, 26–28, 34). Other complications include infection, neurologic deficits, and epilepsy (1). The differences in the incidence of postoperative hemorrhages may be attributed to the heterogeneity in the definitions used. Kulkarni et al. (35) reported that 59.8% (61/102) of patients developed hemorrhages after non-robotic stereotactic biopsy, while the incidence of clinically symptomatic hemorrhage was only 5.8%. In the present study, symptomatic hemorrhages documented on postoperative CT scan were observed in 10 cases (6.62%), including 4 cases (8.51%) in the frame-based group and 6 cases (5.77%) in the robot-assisted group, consistent with the literature. Intratumoral hemorrhage was observed in all cases with no bleeding along the biopsy trajectory. We hypothesize that preoperative high-resolution MRI decreased the risk of injury to sulcal vessels or bridging veins, and the accuracy of the stereotactic tool also played an important role. In this respect, Marc Zanello et al. (36) reported that postoperative intracerebral hematomas were mainly derived from human-related errors during trajectory planning.

Frame-based stereotactic surgery remains the gold standard for biopsy, although it has some drawbacks. First of all, taking the Leksell frame as an example, some patients may find it distressful to place a rigid head frame on their head (18). In contrast, after five skull nails were fixed when using the SINO surgical robot, the patient did not experience any discomfort. In some cases, the skull size can limit the application of the frame, especially in children with small skulls and adults with large skulls. In the robot-assisted group, six patients were under the age of 10, of which the youngest was only 1 year and 3 months old. Compared with the framework, robots may have broader indications in neurosurgical biopsy. Moreover, if the lesion is located at an extreme site, such as the inferior temporal lobe or posterior fossa, frame-based stereotactic biopsy may be challenging (31), and robot-assisted surgery is more convenient in such cases. Compared with other robots, the SINO surgical robot can adopt laser surface registration, skin fiducial registration, and bone fiducial registration. Besides, three-dimensional (3D) visualization technology of craniocerebral vascular can be applied to this robot to show the vascular structure. It can also intelligently perceive obstacles on the path and automatically judge the feasibility of the operation.

## Limitations

Although we expounded on the advantages of robot-assisted biopsy, such as high flexibility, safety, and precision, we acknowledge there were still some limitations in this study. We only summarized and compared the two groups of stereotactic biopsies. At present, the surgical robot has also been applied for stereotactic electrode implantation, deep brain stimulation, and other operations in our center. These cases will be included in our future studies. Moreover, the present study was a single-center retrospective study with small sample size, and hence methodological limitations could not be avoided.

## Conclusions

This study presented the largest series of stereotactic biopsies with SINO surgical robot-assisted platform. Robot-assisted brain biopsy becomes an increasingly mainstream tool in the neurosurgical procedure. Our systematic analysis demonstrated that the SINO surgical robot system is as efficient, accurate and safe as the standard frame-based stereotactic biopsy and provides a reasonable alternative to stereotactic biopsy in neurosurgery. Moreover, compared with the classic framework, the robotics may have wider indications in brain biopsy due to its high flexibility.

## Data Availability Statement

The raw data supporting the conclusions of this article will be made available by the authors, without undue reservation.

## Ethics Statement

The studies involving human participants were reviewed and approved by Chongqing Sanbo Jiangling Hospital Ethics Committee. Written informed consent to participate in this study was provided by the participants' legal guardian/next of kin. Written informed consent was obtained from the individual(s), and minor(s)' legal guardian/next of kin, for the publication of any potentially identifiable images or data included in this article.

## Author Contributions

CL and PC participated in the experimental design. YH collected the data and finished the paper. JP and YL participated in the data collection and post-processing. SC and FL not only provided the experimental equipment and post processing workstation but also revised the first draft of the paper. HZ and AA participated in the data collection and giving a hand in the statistical analysis. All authors contributed to the article and approved the submitted version.

## Funding

This work was supported by Scientific Research Common Program of Beijing Municipal Commission of Education (KM201910025002).

## Conflict of Interest

The authors declare that the research was conducted in the absence of any commercial or financial relationships that could be construed as a potential conflict of interest.

## Publisher's Note

All claims expressed in this article are solely those of the authors and do not necessarily represent those of their affiliated organizations, or those of the publisher, the editors and the reviewers. Any product that may be evaluated in this article, or claim that may be made by its manufacturer, is not guaranteed or endorsed by the publisher.
